# Patient-Centered Care for Patients with Cardiometabolic Diseases: An Integrative Review

**DOI:** 10.3390/jpm11121289

**Published:** 2021-12-03

**Authors:** Maria do Céu Marques, Rute Pires, Miguel Perdigão, Luis Sousa, César Fonseca, Lara Guedes Pinho, Manuel Lopes

**Affiliations:** 1 Nursing Department, Universidade de Évora, 7000-811 Évora, Portugal; mcmarques@uevora.pt (M.d.C.M.); ruteisabelpires@gmail.com (R.P.); mpp_12@sapo.pt (M.P.); lmms@uevora.pt (L.S.); cfonseca@uevora.pt (C.F.); mjl@uevora.pt (M.L.); 2 Comprehensive Health Research Centre (CHRC), Universidade de Évora, 7000-811 Évora, Portugal; 3 Hospital do Espírito Santo de Évora, 7000-811 Évora, Portugal

**Keywords:** patient-centered care, patient care planning, cardiometabolic disease

## Abstract

Patient-centered care is essential in high-quality health care, as it leads to beneficial outcomes for patients. The objective of this review is to systematize indicators for the care of patients with cardiometabolic diseases based on patient-centered care, extending from the stages of diagnostic evaluation and care planning to intervention. An integrative literature review was conducted by searching seven scientific databases, and a narrative analysis was performed. A total of 15 articles were included, and indicators related to diagnosis and care planning/intervention were extracted. In the planning of care centered on the person with cardiometabolic diseases, the individuality, dynamics of the processes, flexibility and the participation of all stakeholders should be taken into account. The needs of the person must be addressed through the identification of problems; establishment of individual goals; shared decision making; information and education; systematic feedback; case management; meeting the patient’s preferences and satisfaction with care; engagement of the family; and therapeutic management. The indicators for intervention planning extracted were behavioral interventions, therapeutic management programs, lifestyle promotion, shared decision making, education patient and information, interventions with the use of technology, promotion of self-management, program using technology, therapeutic relationship, therapeutic adherence programs and specialized intervention.

## 1. Introduction

Patient-centered care (PCC) is defined as care that establishes a partnership among practitioners, patients, and their families to ensure that the care needs, values, and preferences of patients are satisfied [1]. PCC is characterized by empathy, respect, engagement, relationships, communication, shared decision making, holistic approaches, individualized focus and coordinated care [2]. From this perspective, the relationship between the patient and the caregiver is strengthened and is characterized by information sharing, empathy, and empowerment. In the partnerships established, the team’s sensitivity to the patient’s needs and their engagement in care stand out. In health promotion, the following dimensions are essential: case management and patient empowerment [1].

PCC is fundamental in high-quality health care and leads to beneficial outcomes for the patient, such as knowledge about their health, better skills to manage self-care behaviors, increased satisfaction, medication adherence, improved quality of life, and reduced admissions, readmissions and length of hospital stay. Regarding family members, PCC reduces the intensity of stress, anxiety, and depression, increases satisfaction, and improves relationships with health professionals. It also has beneficial effects on the health system, as services with a good cost–benefit ratio are provided [1,3].

For care to be centered on the patient, it is essential for decision making to be shared. In this care model, health communication is central to supporting patient engagement. Shared decision making has evolved greatly in recent years in Europe and North America. It reflects attitudes, beliefs and practices that should be explicit in PCC and that differ among different regions of the world and, on a smaller scale, among the different regions of a country [4].

One of the greatest challenges of the century is to optimize the health and quality of life of the population. The unavoidable reality of the high prevalence of cardiovascular diseases in the adult population and the aging of the population is a problem. Approximately 70% of elderly individuals have two or more chronic diseases, typically including a cardiovascular disease and a metabolic disease, which makes decision making difficult for health professionals and patients. A patient-centered approach and shared decision making can help ensure that the benefits of care decisions outweigh the harms of multiple comorbidities [5].

Cardiometabolic diseases (CMDs) are one of the main causes of comorbidities and death worldwide, both in developed countries and in emerging and underdeveloped economies. It is estimated that approximately 25% of the global adult population suffers from CMDs. Considered by the World Health Organization as the epidemic of the 21st century, CMDs encompass, among other conditions, obesity, diabetes and hypertension, which are considered risk factors for the development of cardiovascular diseases, such as acute myocardial infarction and stroke and peripheral arterial disease [6].

The risk factors for the development of CMDs are diverse but interrelated, such as hypertension, elevated fasting glucose, obesity and elevated triglycerides; understanding these contributes significantly to the development of clinical and/or treatment strategies [7]. However, there are some risk factors that cannot be changed, i.e., unmodifiable, such as older age (increasing age increases risks), genetic predisposition and sex (more frequent in men than in women until the age of menopause). However, in the case of most CMDs, the most common factors are almost always linked to lifestyle and therefore are modifiable, for example, diet, sedentary lifestyle, smoking and alcohol consumption, on which we can act on a day-to-day basis by preventing or treating. The less active a person is and the more foods rich in carbohydrates, sugars, and sodium they consume, such as fast food, cookies and/or sweets, the greater the chance of developing CMDs [6].

To reduce the chances of developing CMDs across the years, it is very important to implement lifestyle changes, such as prioritizing healthy eating and reducing the consumption of simple carbohydrates, sugars, and sodium; exercising at least 150 min per week; controlling alcohol intake; reducing or avoiding smoking; adopting measures that reduce stress (e.g., reading, sports and meditation); and having nights of restful sleep [6].

In this sense, it is essential that health professionals carry out a diagnostic assessment, develop an adequate care plan that is adapted to the lifestyle and environment in which they are inserted, and implement person-centered care. However, the scientific literature is sparse and has little consensus on diagnostic assessment strategies, care planning and interventions centered on people with patients with CMDs.

This integrative literature review aims to identify and systematize indicators for the care of patients with CMDs based on PCC, extending from the stages of diagnostic evaluation and care planning to intervention.

## 2. Materials and Methods

### 2.1. Protocol and Registration

The protocol for this review was registered and published in PROSPERO (CRD42021240880), including the unique characteristics of the methodological procedures, namely, the eligibility criteria and data synthesis strategies.

### 2.2. Study Design

The review was conducted in accordance with the Preferred Reporting Items for Systematic Reviews and Meta-Analyses for Protocols (PRISMA-P) statement [8,9]. Our methods followed the framework of Whittemore and Knafl [10].

The review was designed based on the following research questions:

- How can the diagnostic evaluation of patients with cardiovascular and metabolic disease be performed, either through assessment instruments or other types of evaluation?
- What are the care planning strategies focused on patients with cardiovascular and metabolic disease developed with the objective of promoting health and preventing and/or reducing complications related to the aforementioned diagnoses?
- What intervention strategies aim to implement care processes focused on patients with cardiovascular and metabolic disease?

### 2.3. Search Strategy

To conduct the review, a broad literature search was conducted in the following databases: EBSCOhost Research Platform; CINAHL^®^ Plus with Full Text; Nursing and Allied Health Collection; Cochrane Plus Collection, including Cochrane Central Register of Controlled Trials, Cochrane Database of Systematic Reviews (CDSR) and Database of Abstracts of Reviews of Effects (DARE); MedicLatina; MEDLINE^®^, including International Nursing Index; PubMed via MEDLINE; EMBASE; Scopus; CINAHL; Web of Science; The Cochrane Library (Cochrane Database of Systematic Reviews, Cochrane); and the Virtual Health Library (VHL).

The search strategy was adjusted to each database and was limited to finding the most recent evidence from the last 10 years, dating from 2011 to 2021, including publications in English, Portuguese, Spanish and Italian.

### 2.4. Search Terms and Boolean Operators

The search involved the combination of four basic concepts in line with the medical subject heading (MeSH) terms; the search phrase was as follows: ((metabolic disease) OR (cardiovascular disease)) AND ((patient-centered care) OR (patient care planning)).

First, an exploratory study was conducted without limitations. However, given the large number of results, the search was limited to the title, abstract and/or keywords in the different databases.

### 2.5. Data Collection and Analysis

#### 2.5.1. Selection of Studies

The studies were selected in different phases. Duplicates were removed from the different search engines. Two reviewers independently analyzed the eligibility of studies to reduce bias and did so by reading the title and abstract. Studies that did not meet the inclusion criteria of the review were excluded. A third reviewer was consulted when disagreements or questions emerged. Last, the full text of the articles was evaluated following the same methodology.

#### 2.5.2. Data Extraction

The reviewers who selected the studies also extracted the data independently. The third reviewer helped in cases of questions or disagreements.

A descriptive evaluation of each study was performed using an instrument designed for data extraction, taking into account the defined research questions.

#### 2.5.3. Quality Appraisal

In the evaluation of quantitative, qualitative or mixed studies, it was decided to use the instruments of the Joanna Briggs Institute (JBI), Adelaide, Australia (2020). These instruments rigorously evaluate the essential criteria of primary studies. The critical evaluation instruments were applied by two independent reviewers, with recourse to the third reviewer for consensus in case of disagreement. The result of the critical evaluation of the studies was not defined as an inclusion/exclusion criterion. All studies selected up to this stage were included.

#### 2.5.4. Strategy for Data Synthesis

This review includes articles with different study designs. We chose to perform a structured narrative analysis of the findings to answer the research questions [10].

## 3. Results

**Figure 1:**
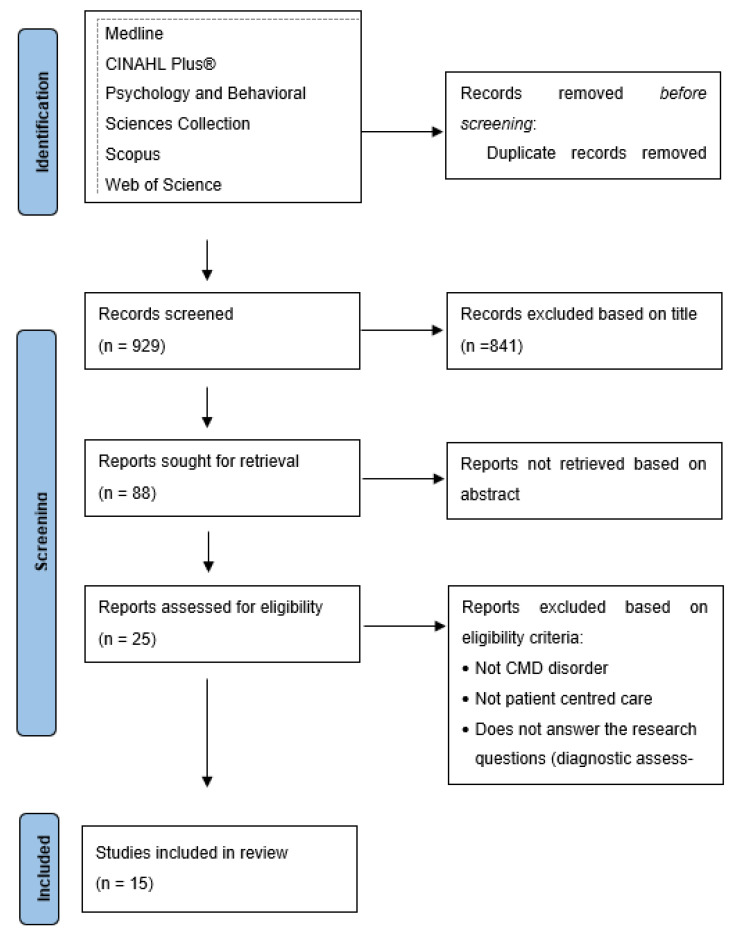
PRISMA flowchart. From: Page MJ, McKenzie JE, Bossuyt PM, Boutron I, Hoffmann TC, Mulrow CD et al. The PRISMA 2020 statement: an updated guideline for reporting systematic reviews. Melbourne, Australia. *BMJ* **2021**, *372*, n71. doi: 10.1136/bmj.n71 [9].

The search generated 946 results accepted for reading of the title. After that, 17 were removed because they were duplicates, and 841 were removed because the title did not fit the topic. The abstracts of the 88 selected articles were read, and the full text of 25 articles was analyzed. After reading the full text and applying the inclusion criteria, 10 articles were eliminated (Figure 1).

Table 1 shows the results of the studies.

**Figure 2:**
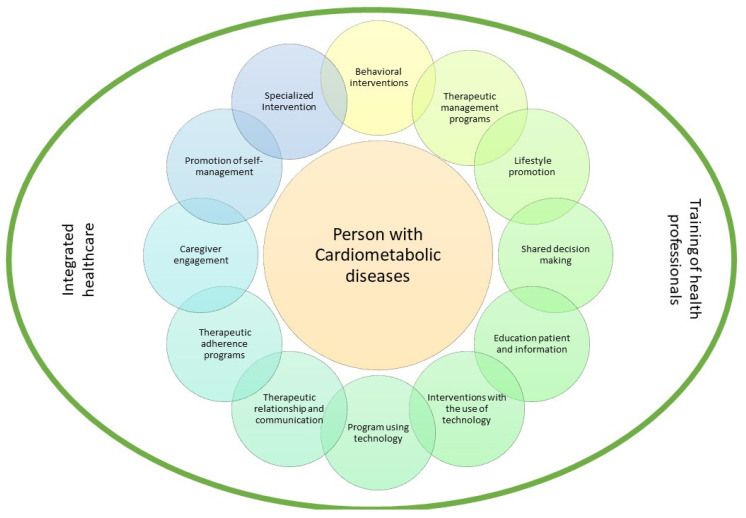
Patient-centered intervention model.

A content analysis was performed based on the extracted indicators and a patient-centered intervention model was built (Figure 2).

The intervention indicators extracted from the studies were: Behavioral interventions [15,21,24,25]; Therapeutic management programs [17,23,24]; Lifestyle promotion [11,13,14,24]; Shared decision making [12,14,16,18]; Education patient and information [15,21,22,24]; Interventions with the use of technology [11,17,25]; Program using technology [11,17,18,24,25]; Therapeutic relationship and communication [13,25]; Therapeutic adherence programs [16,21,23,25]; Caregiver engagement [12,22]; Promotion of self-management [15,21,25]; and Specialized intervention [18,22].

## 4. Discussion

This review integrates studies that allow for performing a narrative analysis of process, outcome and improvement indicators resulting from diagnostic evaluations and care planning and/or interventions in patients with CMDs.

### 4.1. Diagnostic Evaluation

Person-centered care models reinforce the satisfaction of the person’s needs. Regarding diagnostic evaluations, we found several instruments that are conducive to this care model. Most of the studies found based the diagnostic evaluation on person-centered data collection instruments, such as semi-structured interviews with different care stakeholders, including patients, practitioners and/or family members [12,15,21]. The American College of Cardiology and the American Heart Association (2013) published new guidelines to assess the risk of cardiovascular disease; the guidelines are centered on the evaluation of risk scores, providing a prediction of cardiovascular risk in order to identify the main risk factors and risk markers in primary and secondary prevention [16,26]. In the study by Dhukaram, Baber and De Stefanis (2012) [17], the evaluation was performed based on a focus group to understand the concerns and perceptions of patients. In a study conducted in Taiwan, the diagnostic evaluation was performed based on data collected from the National Health Insurance Research Database [20]. In the study by Duda et al. (2013) [25], patient evaluations were performed by a doctor, following the guidelines of the European Society of Cardiology for the medical diagnosis of heart failure (Maggioni et al., 2013) [27]. The American Heart Association Diabetes and Cardiometabolic Health Summit reinforces the importance of evaluating seven health behaviors and cardiometabolic risk factors [28].

Based on the analysis of the various studies, we highlight the importance of conducting an individualized cardiometabolic risk assessment in order to implement primary and secondary prevention strategies adapted to individual risk.

### 4.2. Care Planning and Intervention

The planning of care and the respective intervention found in the selected studies revealed a diversity of data that mostly converge on care centered on patients and their individual conditions. The study conducted by Iturralde et al. (2019) [11] designed an intervention to develop knowledge and skills in patients who were systematically unable to achieve therapeutic management goals and control cardiovascular risk factors. The e-health intervention for patient training and self-management was based on the use of voice or video calls to reinforce self-management behaviors and skills. The authors found improvements in the care process and patient engagement. However, there were no evident improvements in the control of cardiovascular risk factors. In the study by O’Leary et al. (2016) [12], the intervention performed focused on cardiac telemetry monitoring. The use of technology had great relevance in the care process was the improvement of communication between patients and health professionals. However, there were no significant differences in decision making, information sharing or patient satisfaction. The indicator related to communication was also found in the study by Kim and Rich (2016) [18]. According to these authors, discussing the management plan with the patient is important, and providing a written summary can facilitate communication among stakeholders. In the study by McBride et al. (2014) [22] on the management of patients with heart failure, specialist nurses used patient-held alert cards to improve communication and care continuity for these patients. The involvement of specialist nurses facilitates care continuity for patients with heart failure at different levels of care in the health system, as well as safety and efficacy. The alert cards enabled both patients and caregivers to take a more active role in PCC.

Reducing cardiovascular risk is a cross-cutting goal in Western society. Lenz and Monaghan (2011) [13] demonstrated this concern ten years ago. They conducted a study that implemented a program to reduce cardiovascular risk based on an intervention conducted in a community pharmacy setting. They utilized various tools, such as a lifestyle adherence diary, nutrition diary, pedometer, home blood pressure monitor, monthly news bulletin, monthly support group meeting, blog site and educational materials. They observed improved compliance with the guidelines on cholesterol, increased consumption of fresh fruits and vegetables, improved communication between patient and health professionals, and increased awareness and accountability for health. Very positive indicators, such as decreased cardiovascular risk, decreased blood pressure levels, decreased blood glucose levels, and decreased weight, were obtained in that study. At the end of the pilot program, 80% of participants consistently exercised at a level that met the guidelines (>150 min/week) for 3 months. In addition, all participants increased their combined consumption of fresh fruits and vegetables, from an average of 0.9 to 4.5 servings per day. The average weight loss was greater than 12 lbs per person (more than 5% per person); the individual risk of cardiovascular disease improved on average by 34%; and for cardiac diseases only, the individual risk improved on average by 47% after 12 months. For more than half of the participants, communication with health professionals improved. The vast majority considered that the program helped them become more aware of their personal health needs and made them more responsible for actions related to lifestyle [13].

The guidelines issued in 2013 by the American College of Cardiology and the American Heart Association described in the study by Montori, Brito and Ting (2014) [16] showed improvements in the implementation of PCC and shared decision making between patients and health professionals. Additionally, Sassen, Kok, Schepers and Vanhees (2014) [19] conducted a study to test the efficacy of a web-based intervention for the clinical practice of PCC; however, there was no significant difference between the intervention and control groups. The study by Kornelius et al. (2015) [20] revealed that a diabetes shared care program, an integrated care model designed to increase the quality of care for patients with diabetes, had a positive impact. In addition, the outcomes pointed to a lower risk of cardiovascular events and all-cause mortality. Lifestyle improvement recommendations, such as cholesterol reduction, smoking cessation, weight loss, and blood pressure control, are essential in the control of CMDs [14]. The cited study constructed an expert system that learned knowledge on lifestyle and associated cardiometabolic risks from the Atherosclerosis Risk in Communities (ARIC) study data using k-nearest neighbor prediction models. The system collects and analyzes information that improves decision making and patient commitment to adopt a healthy lifestyle.

PCC is associated with less uncertainty in illness than is usual care. Dudas et al. (2013) [25] report that the results of their study suggest that compared with hospitalization-centered care, PCC for patients hospitalized for heart failure seems to have a positive effect by reducing self-reported uncertainty regarding the disease. When the caregivers and patients worked together as partners in a structured PCC plan, many of the issues and uncertainties with which the patients struggled were clarified and resolved during hospitalization. Many patients reported that in the current health care system, they often have to navigate through a fragmented care system where the perspective of the health professional prevails instead of receiving care designed to focus on individual needs, preferences and values. Additionally, PCC reinforced the patients’ perception of the efficacy of treatment. In the multicenter study conducted by Dhukaram, Baber and De Stefanis (2012) [17] on understanding the concerns and perceptions of patients regarding pervasive healthcare systems, patients provided recommendations for system improvements and, as indicators, improvements in health status monitoring as a facilitator of contact between patient and doctor and assurances of confidentiality and privacy with respect to data security.

The interventions studied are highly heterogeneous and centered on recommendations for health promotion and prevention of cardiometabolic risk factors and using information and communication technologies (phone calls, telemedicine, e-health).

In general, the studies included in this integrative review show the following results: improvements in the care process, patient engagement [11], improvements in health status monitoring [17], improvements in communication between patient and health professionals [12,13,17,18,22,25], improvements in shared decision making between patients and health professionals [14,16], data improvements, respecting data privacy and confidentiality [17], care continuity [22], and improved compliance with guidelines and accountability for health [13,14]. These results corroborate previous studies related to patient- and family-centered care with regard to improved knowledge, self-care management, medication adherence and patient satisfaction [3].

The clinical results found in the studies were a decrease in cardiovascular risk factors, such as decreased blood pressure levels, decreased blood glucose levels, decreased weight [13,14,20] and decreased mortality [20]. The evidence found seems to be in agreement with the results previously found by Park et al. (2018) [3] because by reducing the risk factors, there will be better symptom management and better self-care, with a consequent reduction in hospital admissions.

Interventions aimed at patients with cardiometabolic risk can be in-person or remote (e-health) and should be based on the principles of PCC to improve the safety and quality of care. In this sense, the approach to patients should be based on the therapeutic relationship, be holistic and individualized, in which there is empathy, respect, and patient involvement in decision-making, and communication should be clear and assertive. The studies found reported that PCC improved patient assessment, self-care, medication adherence, health and quality of life, and decreased cardiometabolic risk and mortality.

These results are in line with the findings of studies in which educational interventions aimed at lifestyles were carried out with the objective of reducing cardiometabolic risk in a more cost-effective way [29,30].

The main limitation of this study was the decision to conduct the search in electronic databases and portals in Portuguese, English, Italian and Spanish, which may have led to the non-inclusion of publications on the subject in other languages. Another limitation is the heterogeneity of the studies that limits the comparison of findings. However, it allows us to verify the complexity of evaluating patients with CMDs and the variety of interventions in this context.

## 5. Conclusions

We emphasize the importance of PCC models for patients with CMDs, highlighting the importance of performing a periodic evaluation of cardiometabolic risk with special emphasis on lifestyle assessment.

In intervention models focused on patients with cardiometabolic risk, an approach that encompasses behavioral interventions, therapeutic management programs, lifestyle promotion, shared decision making, education patient and information, interventions with the use of technology, promotion of self-management, program using technology, therapeutic relationship, therapeutic adherence programs and specialized intervention.

In care planning, some technological strategies can improve the care process and the outcomes, such as the use of e-health programs, telemedicine, follow-up through phone calls, the use of lifestyle diaries, and the use of information systems that support shared decision making.

In addition, PCC are related to improvements in communication, patient–practitioner relationships, shared decision making, medication adherence, engagement and responsibility for health, and self-care and care continuity. With regard to clinical outcomes, the PCC approach allows for improving health and quality of life by reducing cardiometabolic risk factors, thus translating into reduced mortality and a decreased need for hospitalization, and hence the associated costs.

## 6. Content Analysis

We find that based on the extraction of results as part of the literature review process, there are different interventions/strategies focused on patients with cardiovascular and metabolic diseases that have a significant therapeutic impact on the quality of the care process, with reductions in health complications related to the aforementioned diagnoses and health gains, thus contributing to the effective monitoring of cardiovascular risk and positive outcomes of people-centered care through coordination, collaboration and partnership.

## Figures and Tables

**Table 1:** Study results.

Aim/Hypothesis	Author	Year	Study Type	Intervention/Strategy	Results/Conclusions
A behavioral intervention to increase patient activation, patient-centered care processes, and healthcare system engagement would lead to improved CVD risk factor control	Iturralde, Sterling, Uratsu, Mishra, Ross and Grant [11]	2019	Randomized controlled trial	CREATE Wellness was an intervention designed to develop knowledge and skills in patients systematically unable to achieve the therapeutic goals of management and control of CVD risk factors. This consisted of three group-based patient activation sessions two weeks apart that included between-session contacts with the interventionist (i.e., text message, voice or video call) to reinforce self-management behaviors and skills.	There were reports of improvements in the patient-centered care process. The intervention group was more frequently involved with the health system using online tools. The intervention increased patient’s engagement with the health team. Despite these significant changes, CVD risk factor control did not improve compared to control patients in response to the intervention, highlighting the challenge of improving CVD risk reduction among patients persistently not meeting therapeutic goals.
To evaluate the effect of patient centred bedside rounds (PCBRs) on measures of patient-centred care	O’Leary, Killarney, Hansen, Jones, Malladi, Marks and Shah [12]	2016	Cluster randomized controlled trial	Semi-structured interviews with input from patients, family members and frontline professionals were conducted at Northwestern Memorial Hospital (NMH) in units equipped with cardiac telemetry monitoring and with implemented daily interprofessional bedside rounds.	After discharge, there was no difference in patients’ perceptions of whether nurses and doctors worked as a team or whether the staff included them in treatment decisions. The intervention, designed in collaboration with the patients and their family members, had no impact on patients’ perception regarding shared decision making, activation or satisfaction with care, calling into question the intervention as a strategy to improve patient-centered care. More research studies are needed to identify approaches that can be implemented in hospital settings to improve patient-centered care.
To describe a patient-centered medication therapy management (MTM) program that focuses on lifestyle medicine.	Lenz and Monaghan [13]	2011	Quasi -experimental	A program was implemented to reduce cardiovascular risk based on the intervention of a community pharmacy located in Omaha, NE. The program used various tools (i.e., lifestyle adherence diary, nutrition diary, pedometer, home blood pressure monitor, monthly news bulletin, monthly support group meeting, blog site, and educational materials) to improve patient-centered care and patient outcomes.	Most participants, regardless of hypertension diagnosis, had improvements in blood pressure values, met cholesterol guidelines, lowered blood glucose levels, met exercise guidelines (>150 min/week), increased their combined intake of fresh fruits and vegetables, and lost weight. The individual CVD risk improved on average by 34%, and for heart diseases only, it improved on average by 47% after 12 months. More than half of the participants improved their communication with other health professionals. The vast majority commented that the program helped them become more aware of personal health needs and made them more responsible for actions related to lifestyle.
The authors propose a proof-of-concept machine-learning expert system that learned knowledge of lifestyle and the associated 10-year cardiovascular disease (CVD) risks from individual-level data (i.e., Atherosclerosis Risk in Communities Study, ARIC).	Chi, Street, Robinson and Crawford [14]	2012	Qualitative Descriptive	Construction of an expert system that learned knowledge of lifestyle and associated CDV risks from the data from the Atherosclerosis Risk in Communities (ARIC) study using k-NN prediction models.	The results showed that the optimal individualized, patient-centered lifestyle consistently reduced the 10-year CVD risk. The top three recommended lifestyles were reduce cholesterol intake, quit smoking, and lose weight. The majority (54.4%) of smokers received recommendations to quit smoking, 37.2% received recommendations to control their cholesterol, and 4.8% received recommendations to control their weight (BMI). Clinically, weight control was usually recommended for diabetes and hypertension patients, and weight control was indeed the first recommendation for diabetes patients and the second recommendation for hypertension patients. In addition, the system showed that lowering cholesterol was among the top two recommendations for both diseases. It is expected that this interactive decision process (among patients, physicians and the system) will increase patients’ involvement and participation and subsequently may improve an individual’s commitment to a healthy lifestyle.
To explore how clinicians deliver patient-centred care for women (PCCW), challenges they face, and the strategies they suggest are needed to support PCCW.	Filler, Dunn, Grace, Straus, Stewart and Gagliardi [15]	2020	Qualitative	Semi-structured interviews with clinicians from the province of Ontario, Canada, whose script consisted of three questions: (1) How do you tailor patient-centered care for women? (2) What factors challenge patient-centered care for women? (3) What strategies or interventions would help you deliver or achieve patient-centered care for women?	Clinicians emphasized that women face unique challenges in seeking health care and communicating about health care issues that warrant tailoring of PCC. Approaches used by clinicians to adapt PCC to women were: fostering a healing relationship, exchanging information, addressing emotions/concerns, managing uncertainty, decision making, and allowing self-management. Participants reported that offering flexible options for follow-up appointments was essential to monitor the response to treatment and health status.
To apply the ACC/AHA guidelines in a patient centered and practical perspective, 3 cases illustrate the evidence-based approach espoused by the new guidelines, with 1 important modification	Montori, Brito and Ting [16]	2014	2 Cases/Opinion	In 2013, the American College of Cardiology and the American Heart Association (ACC/AHA) published new guidelines for assessing CVD risk and for the treatment of high blood cholesterol levels to reduce CVD. These new guidelines replaced the Adult Treatment Panel III (ATPIII) guidelines for the detection, evaluation, and treatment of high blood cholesterol that guided clinical practice for more than a decade. The new guidelines divert focus from lowering low-density lipoprotein (LDL) cholesterol levels to treating CVD risk and therefore are no longer pure cholesterol guidelines and discourage the prescription of lipid-lowering medications. To demonstrate the ACC/AHA guidelines in a patient-centered perspective, three cases were presented.	Clinicians who consider applying the guidelines should determine the 10-year CVD risk for each patient and engage the patient in shared decision making using evidence-based approaches. The new ACC/AHA guidelines therefore create an opportunity to advance patient-centered care and shared decision making. Rather than routinely prescribing statins to the millions of adults who have a 10-year CVD risk of at least 7.5%, the realization of this opportunity requires clinicians to engage in deliberation with individual patients about the potential benefits, harms, and burdens of statin use.
To examine patient and caregiver perceptions of this technology to further develop an understanding of the benefits and functionalities that prospective patients deem as desirable, undesirable, inadequate or in need of further development.	Dhukaram, Baber and De Stefanis [17]	2012	Focus group studies	Focus group interviews and its results were used to understand patients’ concerns and perceptions toward pervasive healthcare systems and to explore potential barriers to the acceptance of the BraveHealth system, with participants recruited in Italy and England. After the presentation of the components of the system, the patients were asked to answer questions about the usability of biomedical devices, wearable units, touch screen technologies and virtual communities.	ITALY All participants had previously used biomedical devices; they suggested a system they can wear and switch on or off manually. The device they imagined should even notify its state of functioning, making them aware if it is charged and situated correctly on the body. All the patients asked for the implementation of a button to warn doctors or caregivers about emergencies. Three of those interviewed would like a fully integrated unit, i.e., as part of their clothing. ENGLAND Overall, participants in these focus groups showed a positive response in regard to the potential benefits of the BraveHealth concept. In general, these responses were related to the benefits of real-time monitoring of a range of parameters and the capability to receive a quick response to potential problems. However, concerns remained about reliability, security, privacy and trust.
The Canadian Journal of Cardiology	Kim and Rich [18]	2016	2 Cases/Opinion	Case Study	A disease management program led by a heart failure nurse specialist and involving close telephone follow-up and home visits may reduce heart failure and all-cause admissions and mortality. Older patients with mild-to-moderate frailty may benefit more from such a program. The rate control strategy in atrial fibrillation was associated with fewer adverse events and admissions than was the rhythm control strategy, without differences in mortality, cardiovascular events, or quality of life. To reduce the treatment burden and improve adherence, medication dosing should be changed to once daily, if possible. Importantly, the CVD management strategy should be part of the general practitioner’s overall management plan, which should also address the patient’s non-cardiovascular conditions. In discussing the management plan with a patient, it is important to ensure that the plan is consistent with the preferences of the patient and that the patient agrees with the recommendations. A written summary can facilitate clear communication of the rationale for and details of the management plan with the family and other members of the healthcare team. The management plan should be revised based on the prognosis, functional status, and personal preferences of the patient. Discussion of advance care planning should take place before cognitive impairment progresses. Cardiologists should work closely with general practitioners to adopt a patient-centered approach to manage CVD and non-cardiovascular health that will have the greatest impact on functioning and quality of life in older adults with CVD and multimorbidity.
To increase health care professionals’ intention and encouraging behavior toward patient self-management, following cardiovascular risk management guidelines.	Sassen, Kok, Schepers and Vanhees [19]	2014	Experimental	The effectiveness of a Web-based intervention in the clinical practice of patient-centered care was tested. The intervention was developed to optimize processes of shared decision making and self-management, following the protocol for intervention mapping. The objective was to increase health care professionals’ intention and behavior toward encouraging patient self-management. Participants were health care professionals with at least a bachelor’s degree in nursing or physiotherapy and who had regular consultations with patients with cardiovascular risk factors. All participants were offered a three-hour training session.	Professionals in the intervention group stated that 59% of their consultation time was devoted to health education. The module to improve professionals’ behavior to optimize processes of shared decision making and self-management was used by 45% of the professionals; 45% used the screen to encourage a patient to think about their personal risk; 39% provided attitudinal change and outcome expectations; 30% provided guidance for resistance to social pressure and seeking support; 28% encouraged subskill enactment. For 24% of the patients, the professional provided planning for behavioral changes; 19% provided guidance regarding putting behavior changes into practice; and 15% provided guidance regarding maintaining behavior changes. Only in one of every five patients was the guidelines for cardiovascular risk management used. Professionals in the intervention group experienced more barriers to encouraging patients than did professionals in the control group.
To evaluated whether participating patients had reduced risks of cardiovascular events, including coronary heart disease, stroke, and all-cause mortality.	Kornelius, Chiou, Yang, Lu, Peng and Huang [20]	2015	Retrospective cohort study	The Diabetes Shared Care Program (DSCP), an integrated diabetes care model designed to increase the quality of diabetes care in Taiwan, was analyzed. Data were obtained from the National Health Insurance Research Database of Taiwan. DSCP participants received integrated care from a physician, diabetes educator, and dietitian.	Compared to nonparticipants, DSCP participants had significantly lower risks of overall CVD events. Patients with a history of hypertension and chronic lung diseases had a higher risk of CVD events. At the end of the study, the provision of integrated care arrangements for diabetic feet had a positive impact on the knowledge of primary care staff and on patients’ attitudes, resulting in an increased number of appropriate referrals to acute specialist services. Diabetic patients with a lower monthly income also had a higher risk of CVD events. Participation in the DSCP was associated with lower risks of overall CVD events, including stroke and all-cause mortality.
To gain insight into what motivates older people living in the community to partake in a cardiovascular prevention programme, and reasons for subsequent continuation or withdrawal.	Ligthart, Eerenbeemt, Pols, Van Bussel, Richard and Van Charante [21]	2015	Qualitative	The sample consisted of PreDIVA participants (all people aged 70–78 years were invited to participate through a letter from their general practitioner). Semi-structured interviews were conducted in six different healthcare centers.	Almost all participants emphasized the importance of the relationship with the practice nurse. In general, participants wished to be involved in medical decisions, such as starting medication or getting additional diagnostic tests, but they were prepared to follow the advice of their health professional, even if they did not always fully understand the rationale. Some interviewees expressed the importance of their autonomy being respected; the perception of being ‘checked up on’ gave a sense of control, safety or being looked after. It was seen as an essential component of the program and a reason for starting and continuing participation. The personal approach of the practice nurse appeared to be crucial. In addition to the nurses’ medical expertise, their ability to listen and to build a personal relationship was strongly linked to trust and prolonged participation. Participants wanted nurses to have a coach-like and supportive attitude.
To explored the potential of patient-held alert cards to improve communication and continuity of care for heart failure patients moving between CHFSNs and hospital settings	McBride, Burey, Megahed, Feldman and Deaton [22]	2014	Qualitative Mixed Approach	This study investigated the management of patients with heart failure (HF) by community heart failure specialist nurses (CHFSNs) by exploring the potential of patient-held alert cards to improve communication and continuity of care for patients with HF. The follow-up was 12 months. Alert cards were issued to 119 patients. The CHFSNs excluded patients with cognitive impairments or frailty.	The involvement of CHFSNs facilitated the continuity of care for HF patients at different levels and sectors of the healthcare system, in addition to improving safety and effectiveness. The alert cards empowered both patients and caregivers to take a more active role in their care. First, although CHFSNs explained an HF diagnosis as part of their assessment, the card itself appeared to prompt patients to ask questions about their condition. In the absence of immediate technological solutions to bridge the primary and secondary interface, this study illustrated how a patient-held alert card can provide informational continuity, as it encouraged general wards to liaise with specialist nurses on admission to ensure the continuation and appropriateness of care.
The impact of a pharmacy-managed program for providing education and discharge instructions for patients with heart failure (HF) was evaluated.	Warden, Freels, Furuno and Mackay [23]	2014	Quasi-experimental Therapeutic Reconciliation	Intervention performed with adult patients admitted to Oregon Health and Science University’s cardiology unit with systolic HF exacerbation as their primary diagnosis. The HF-MED study evaluated the impact of pharmacist involvement in providing education and discharge instructions. Eighty-two percent of patients had an ejection fraction of <30%, 88% had functional class III or IV HF based on the New York Heart Association criteria, and 99% had functional class C or D HF based on the American College of Cardiology-American Heart Association criteria.	The HF-MED program was associated with a significant difference in favor of the pharmaceutical intervention group for both analyzed central measures of the Joint Commission, with adherence that exceeded their respective UHC reference parameters mentioned previously and 30-day readmissions for all causes. There was also a nonsignificant trend in reductions in 30-day readmissions related to HF. When evaluating the experience of patients with the service, 86% believed that medication counseling was important, 91% were satisfied with the service, and 74% were more likely to return to OHSU because of this program.
To evaluate the impact of an intensive, evidence-based preventive cardiology programme on medical and lifestyle risk factors in patients at high risk of developing cardiovascular disease (CVD)	Gibson, Flaherty, Cormican, Jones, Kerins, Walsh, Costello, Windle, Connolly and Crowley [24]	2014	Descriptive	The impact of an intensive evidence-based preventive cardiology program (Croi MyAction program-London) on medical and lifestyle risk factors in patients at high risk of developing CVD was evaluated. All participants were invited to bring a partner to the program, with an uptake rate of 61% among those who had a partner.	Among diabetic patients, glycemic control improved during the program. The prescription of all cardioprotective medications, with the exception of beta-blockers, increased significantly during the program. In addition to biomedical improvements, patients derived significant psychosocial benefits from the program, with fewer patients having raised levels of anxiety or depression at the end of the program (after one year). Participants in the current study completed a program that involved risk factor reduction through lifestyle modification, supplemented by target-driven pharmacological interventions. High cessation rates were observed among smokers, there was increased adherence to the cardioprotective diet, and physical activity increased with an associated improvement in physical fitness. Reductions in BMI and abdominal circumference were observed. Improvements were observed in blood pressure and glycemic control and in all blood lipid fractions.
To evaluate whether PCC is associated with less self-reported uncertainty in illness compared with usual care in patients hospitalized for worsening CHF.	Dudas, Olsson, Wolf, Swedberg, Taft, Schaufelberger and Ekman [25]	2013	Experimental	The study evaluated whether person-centered care with less uncertainty in illness was comparable to usual care (UC) in patients hospitalized for worsening chronic heart failure (CHF). All patients with a prior diagnosis of CHF admitted to five designated wards at the Department of Medicine at Sahlgrenska University Hospital/Östra in Gothenburg, Sweden, were screened for symptoms of worsening CHF (mainly dyspnea and/or fatigue). The patients were assessed by a physician before study inclusion, following the ESC guidelines for diagnosing CHF.	The results of the study suggest that compared to UC, PCC in patients hospitalized for worsening CHF seems to have a positive effect in reducing self-reported uncertainty in illness. When caregivers and patients worked together as partners in a structured PCC plan, many of the issues and uncertainties with which the patients struggled were cleared up and resolved during hospitalization. Many patients reported that in today’s healthcare system, they often have to navigate through a fragmented healthcare system where the perspective of the health professional prevails, instead of receiving care designed to focus on individual needs, preferences and values. PCC strengthened the patients’ perception of treatment effectiveness.

## Data Availability

Not applicable.
